# IL27 controls skin tumorigenesis via accumulation of ETAR-positive CD11b cells in the pre-malignant skin

**DOI:** 10.18632/oncotarget.12581

**Published:** 2016-10-12

**Authors:** Denada Dibra, Abhisek Mitra, Melissa Newman, Xueqing Xia, Camille Keenan, Jeffry J. Cutrera, J. Michael Mathis, Xiao-Jing Wang, Jeffrey Myers, Shulin Li

**Affiliations:** ^1^ Department of Pediatrics, The University of Texas MD Anderson Cancer Center, Houston, TX 77030, USA; ^2^ Banfield Pet Hospital, Sugar Land, TX 77479, USA; ^3^ The University of Texas Health Science Center at Houston, Medical School, Houston, TX 77030, USA; ^4^ Department of Comparative Biomedical Sciences, Louisiana State University School of Veterinary Medicine, Baton Rouge, LA 70803, USA; ^5^ Department of Pathology, University of Colorado Anschutz Medical School, Aurora, CO 80045, USA; ^6^ Department of Head and Neck Surgery, The University of Texas MD Anderson Cancer Center, Houston, TX 77030, USA

**Keywords:** ET1/ETAR, IL27, IL27RA, KRAS, inflammation

## Abstract

Establishment of a permissive pre-malignant niche in concert with mutant stem are key triggers to initiate skin carcinogenesis. An understudied area of research is finding upstream regulators of both these triggers. IL27, a pleiotropic cytokine with both pro- and anti-inflammatory properties, was found to be a key regulator of both. Two step skin carcinogenesis model and K15-KRAS^G12D^ mouse model were used to understand the role of IL27 in skin tumors. CD11b^−/−^ mice and small-molecule of ETAR signaling (ZD4054) inhibitor were used *in vivo* to understand mechanistically how IL27 promotes skin carcinogenesis. Interestingly, using in vivo studies, IL27 promoted papilloma incidence primarily through IL27 signaling in bone-marrow derived cells. Mechanistically, IL27 initiated the establishment of the pre-malignant niche and expansion of mutated stem cells in K15-KRAS^G12D^ mouse model by driving the accumulation of Endothelin A receptor (ETAR)-positive CD11b cells in the skin—a novel category of pro-tumor inflammatory identified in this study. These findings are clinically relevant, as the number of IL27RA-positive cells in the stroma is highly related to tumor de-differentiation in patients with squamous cell carcinomas.

## INTRODUCTION

Mouse models of skin carcinogenesis are similar to human skin cancers, and these models have been important for understanding the etiology of cancer as a step-wise process that includes many facets, such as chronic inflammation, angiogenesis, and stem cell proliferation [1]. Chronic inflammation characterized by immune cell infiltrates and their cytokine release is a common manifestation in skin tumors. Interleukin (IL) 23 promotes epithelial carcinogenesis by reducing CD8 T cell infiltration [2]. Tumor Necrosis Factor (TNF) is another promoter in this model [3]. Immunoglobulin (Ig) deposition in the skin is also a common occurrence in the premalignant skin, and B cell-derived Ig deposition in the skin acts as an immune modulator by sustaining inflammation through activation of Fcγ receptors on resident or recruited myeloid cells [4, 5].

In addition to chronic inflammation, oncogenic mutations acquired by hair follicle stem cells can induce squamous cell carcinoma (SCC) development [6, 7]. Recent studies discovered that these follicular stem cells serve as the cell of origin mainly when the cells go from quiescence into a burst of proliferation during the transition from telogen to anagen phases [7]. However, these stem cells retain their quiescent state and are unable to initiate tumorigenesis even if they gain oncogenes such as KRAS or lose tumor suppressors such as p53 [7]. This study suggests that oncogene gain or tumor-suppressor loss is not sufficient to overcome quiescence and initiate tumorigenesis, and interactions with the microenvironment might be needed.

The link between angiogenesis and stemness requires intensive investigation, as this link has been seen in many cancers of different origins including brain, colon, and skin [8–10]. One mechanism through which the vascular niche promotes tumor initiation or growth is propagation of stem cells [9]. Indeed, Beck et al. showed that VEGF plays a dual role in skin cancers [9]. VEGF signaling in endothelial cells increases vessel density and concomitantly increases the stem cell pool. Additionally, VEGF signaling in an autocrine loop via Nrp1 receptor in stem cells raises VEGF's ability to induce stemness of these cells [9]. While oncogenic gain, stemness, and angiogenesis are all important processes, the molecules which govern the pre-malignant milieu, angiogenesis, and oncogenic stem cells are not well understood.

The cytokine IL27 has pleiotropic functions, including pro- and anti-inflammatory properties in inflammatory and infectious diseases, but the function of IL27 during initiation of skin cancer is obscure [11]. Here we address this unmet need and show for the first time that IL27 promotes tumorigenesis by sustaining a pro-malignant niche characterized by increase in vessel density through ETAR-positive CD11b cells. Additionally, IL27-driven pre-malignant niche in the presence of benzoyl peroxide promotes activation of K15-driven harboring oncogenic KRAS to form tumors, an ETAR signaling-dependent process.

## RESULTS

### IL27 signaling in bone marrow derived cells promotes skin carcinogenesis

To examine more directly the role of IL27 during the early stages of epithelial cancer formation, the well-established two step skin carcinogenesis model (DMBA followed by the promoter benzoyl peroxide (BP) as a promoter) was used. BP rather than TPA was used as a promoter, because C57Bl/6 mouse strain is more susceptible to tumor formation using this promoter as previously delineated by Slaga *et al* [12]. Another reason is that benzoyl peroxide is a free radical generating compound that is used extensively in the pharmaceutical and cosmetic industry [13].

**Figure 1:**
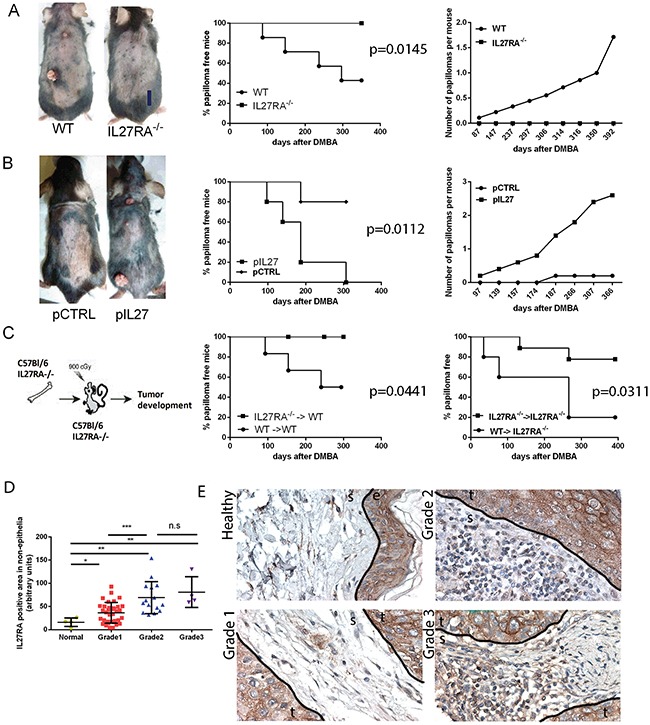
IL27 signaling promotes skin carcinogenesis **A.** IL27RA^−/−^ mice are resistant to cutaneous chemical carcinogenesis when compared to wildtype mice (photograph, left) as they have reduced incidence of papilloma formation over time (middle) when compared to wildtype mice and less papillomas per mouse when compared to control (right). N=8-10. Experiment repeated 3 times. p=0.0145 **B.** Gene therapy administration of plasmid IL27 enhances cutaneous chemical carcinogenesis in C57Bl/6 mice when compared to control plasmid (pCTRL) (photograph, left) as these mice have higher incidence of papilloma formation over time (middle) and higher number of papillomas per mouse when compared to mice treated with control plasmid (right). N=5. Experiment repeated twice. p=0.0112 **C.** Mice lacking IL27RA^−/−^ in bone marrow cells are more resistant to cutaneous chemical carcinogenesis tumor development that mice with wildtype bone marrow cells irrespective of IL27RA in the non-bone marrow cells. P=0.0441 (left) and p=0.0311 (right) (N=6-9, repeated twice with similar results, WT (donor) → IL27RA^−/−^ (recipient)). **D, E.** Quantification and representative photomicrographs of IL27RA positive cells in non-epithelial (stroma) compartment of skin derived from normal patients, or patients with SCC at different grades. E- epidermis, D- dermis, S- stroma, T- tumor.

Interestingly, the lack IL27 signaling (IL27RA^−/−^) during the initiation of carcinogenesis reduced the incidence of papilloma formation over time when compared to wildtype mice (Figure 1A). To further understand whether IL27 can promote tumor incidence in the skin, we administered plasmid DNA into the rear tibialis muscle followed by electroporation as a gene delivery method. This method is efficacious (Supplementary Figure S1) and has often been widely used to induce systemic and continuous supply of IL27 protein in the bloodstream of these mice [14]. As expected, wildtype mice treated with IL27 via gene therapy had a higher incidence and developed more papillomas when compared to control counterparts (Figure 1B). IL27-induced papilloma initiation was compromised in IL27RA^−/−^ mice (Supplementary Figure S2), suggesting that IL27 signaling through its receptor, IL27RA, is needed to promote papilloma initiation. Interestingly, the number of papillomas developed in the wildtype mice treated with control DNA followed by electroporation were lower when compared the wildtype mice there were not treated with any DNA (the administration of DMBA and BP were similar between these mice). Most likely this phenomenon is due to the immune-stimulatory properties of plasmid DNA as our group and others has previously documented that empty control DNA can induce IFN-related genes [15, 16].

Because IL27RA is expressed in both epithelial and immune cells, we used bone marrow transfer to tease out where IL27 signals. The reconstitution efficacy in the peripheral blood between wildtype and IL27R^−/−^ was determined in donors via flow cytometry 8 weeks post bone marrow transfer. The reconstitution was effective and no significant differences were seen in the pheripheral blood prior to skin tumorigenesis treatment amongst wildtype or IL27RA^−/−^ donors (Supplementary Figure S3).

Bone marrow transfer studies showed that IL27 signaling in hematopoietic cells is needed to drive skin carcinogenesis (Figure 1C). Although not reaching statistical difference (P= 0.1224), a trend was observed the recipient mice seemed to also make a difference: wildtype recipient mice are more resistant to tumor formation than IL27RA^−/−^ mice regardless whether they received bone marrow derived from wildtype or IL27RA^−/−^ mice. This observation suggests that IL27 signaling in epithelial cells is protective may play a protective role, but overall effects of IL27 are pro-tumorigenic.

In order to understand the significance and relevance of IL27 signaling in the bone marrow cells to human cancer patients, we analyzed the levels of IL27RA in normal vs. malignant tissue and determined the IL27RA-positive cells in the stroma in correlation to the disease progression. Consistent with the data from the murine model, IL27RA levels in the stroma layer are low in the normal human skin, and the number of IL27RA-positive stromal cells increase as the tumors de-differentiate (grade increases) (Figure 1D, 1E). Meanwhile, IL27RA levels had no correlation to disease stage (Supplementary Figure S4). Therefore, here we establish a direct positive correlation between tumor grade and the number IL27RA-positive stroma cells in the human skin SCC.

### IL27 induces angiogenesis in the pre-malignant skin

**Figure 2:**
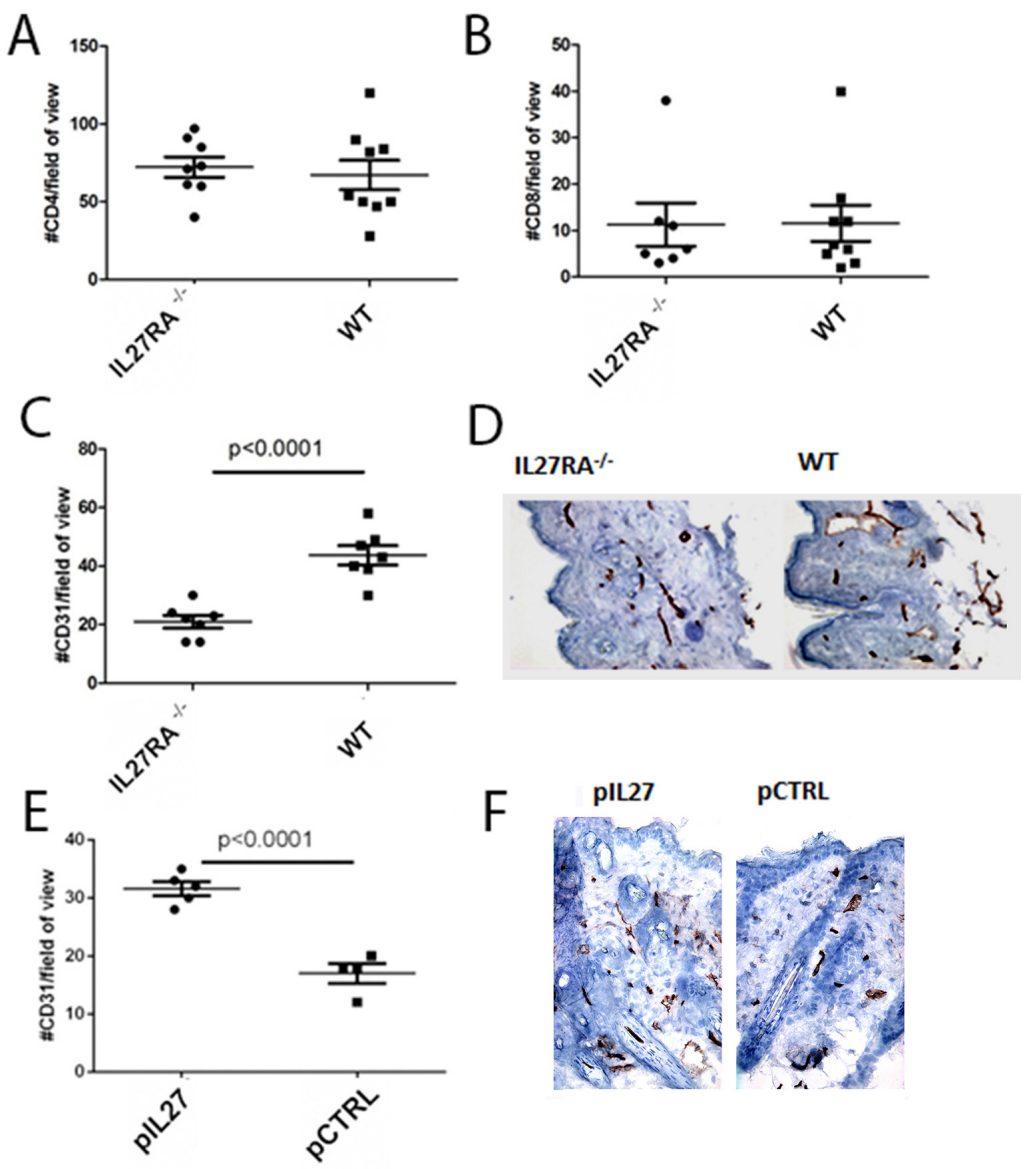
IL27 induces angiogenesis in the pre-malignant skin **A, B.** Similar number of CD4 (A) or CD8 (B) T cells accumulation in the dermis of wildtype or IL27RA^−/−^ 8 months post treatment with DMBA/BP. **C, D.** IL27RA^−/−^ had significantly lower vessel density (CD31) in the dermis when compared to wildtype ones 8 months post treatment. **E, F.** Gene therapy administration of plasmid IL27 significantly enhanced vessel density (CD31) in the dermis when compared to control ones. Photomicrographs were taken at 200X. *, p <0.05.

Changes in the premalignant skin such as increased inflammatory cells infiltration are important factors in skin carcinogenesis. No changes in the number of CD4 or CD8 cells were seen after treatment with IL27 (Figure 2A–2B). Aside from increase in inflammatory cells, angiogenesis can accelerate tumor formation in the skin, and deletion of VEGF reduced skin tumor development, while overexpression of VEGF accelerated papilloma formation [17]. Considering that IL27 promoted tumor initiation in this skin model, we sought to determine how angiogenesis was also affected. Indeed, vessel density was reduced in IL27RA^−/−^ mice when compared to wildtype counterparts (Figure 2C–2D), while exogenous IL27 treatment increased vessel density in the premalignant skin in these mice (Figure 2E–2F). Additionally, the lack of IL27RA in bone marrow derived cells lowered CD31 levels in the skin, suggesting that IL27 signaling in bone-marrow derived cells was needed to promote angiogenesis (Supplementary Figure S5). Notably, IL27 can promote angiogenesis as soon as 3 weeks after-treatment, suggesting that IL27 favors a pre-malignant niche (data not shown).

### CD11B-positive cells are required for IL27 mediated pre-malignant niche formation and skin tumor formation

**Figure 3:**
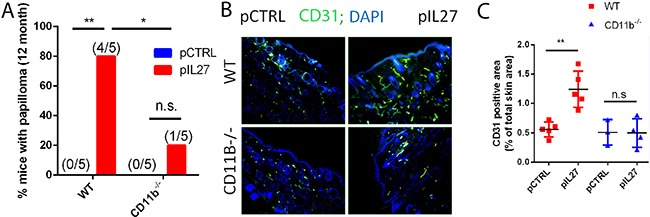
CD11b cells are required for IL27-mediated skin tumor formation and pre-malignant niche formation **A.** IL27-treated CD11B^−/−^ mice are resistant to cutaneous chemical carcinogenesis when compared to IL27-treated wildtype mice (N=5). Chi square was used to determine the differences between the groups. **B, C.** Immunofluorescence (B) and quantification analysis (C) revealed that IL27-mediated increase in vessel density in the skin of wildtype mice was absent in the skin of IL27- treated CD11b^−/−^ mice when compared to control in the pre-malignant skin. Skin tissue was analyzed 3 weeks post IL27 treatment. Photomicrographs were taken at 200X *, p <0.05.

The observation that mice are resistant to cutaneous skin carcinogenesis upon deletion of IL27RA in hematopoietic compartment suggests that most likely IL27 signaling through these cells is needed to promote tumor growth and angiogenesis (Figure 1C). Since IL27 can signal through multiple cell types including myeloid cells [11, 18], comparing the activity of IL27 in the DMBA/BP skin carcinogenesis protocol in CD11b^−/−^ with wild type (WT) mice would reveal the roles of these cell types. Furthermore, CD11b is an important receptor that myeloid cells use in attachment after recruitment to the sites of inflammation [19]. Indeed, IL27-treated mice lacking CD11b^−/−^ cells are ~4 times less likely to develop skin tumors when compared to wildtype mice (Figure 3A). Interestingly, a deficiency of CD11b cells reversed the ability of IL27 to induce angiogenesis in the pre-malignant skin (3 weeks post first IL27 treatment) (Figure 3B, C). Collectively, these data demonstrate that IL27 signaling through CD11b-positive cells are crucial for IL27-mediated promotion of angiogenesis, and skin tumor formation.

### CD11b-positive cells are required for IL27-induced angiogenesis via upregulation of Endothelin Receptor A in the pre-malignant niche

**Figure 4:**
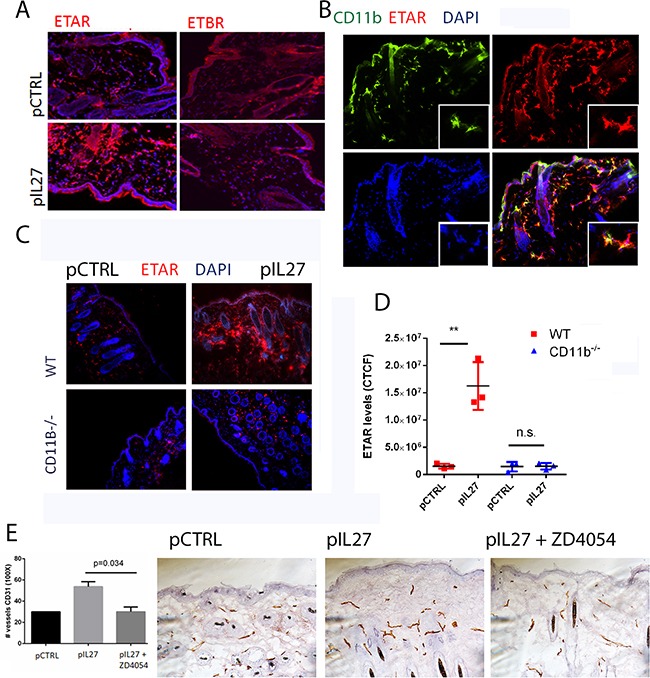
CD11B cells are required for IL27-induced angiogenesis via upregulation of Endothelin Receptor A in the pre-malignant niche **A.** Immunofluorescence analysis showed that IL27 increases Endothelin A receptor levels but not Endothelin B receptor levels in the skin. (B) Immunofluorescence analysis showed ETAR co-stained with Cd11b marker in the skin. Nuclear staining was visualized with DAPI. **B, C.** Immunofluorescence (B) and quantification analysis (C) revealed that IL27-mediated increase in ETAR levels in the pre-malignant skin of wildtype mice was absent in the skin of IL27- treated CD11b^−/−^ mice when compared to control ones. Skin tissue was analyzed 3 weeks post IL27 treatment. **E, F.** Immunohistochemistry analysis showed that the zibotentan (ZD4054) treatment for 3 weeks reversed IL27-mediated increase in vessel density in the premalignant skin (N=3 from independent mice). Photomicrographs were taken at 200X. *, p <0.05.

This study is the first one to show that IL27 promotes skin carcinogenesis and angiogenesis via CD11b cells. One prominent question is mechanistically how IL27 signaling through CD11b-positive cells affects angiogenesis and tumor formation. Upon screening different angiogenic markers (Supplementary Figure S6A), we found that ETAR levels were mostly up regulated by IL27 (Figure 6A), while no changes were observed in Endothelin B receptor levels (Figure 4A). ETAR expression was upregulated in CD11b-positive cells (Figure 4B) but not in the smooth muscle cells (Supplementary Figure S7). Indeed, Endothelin family is often found upregulated in tumors and it promotes angiogenesis. Furthermore, the levels of the ligand for this receptor were elevated in IL27- treated skin (Supplementary Figure S6B). Interestingly, IL27's ability to increase ETAR levels in the premalignant skin is dependent upon CD11b cells; in the absence of these cells IL27 is unable to accrue ETAR-positive cells (Figure 4C, D). Because IL27 regulates the pre-malignant niche in as few as 3 weeks, we sought to determine whether IL27 affects regulates angiogenesis via ETAR signaling. Time-course studies showed that ETAR levels in the skin were induced as early as 72 hour after IL27 treatment while no changes in vessel density were evident at this point (Supplementary Figure S4C-E). These results show that IL27 induces ETAR prior to angiogenesis. To understand whether IL27 affects angiogenesis via ETAR, we used ZD4054, a chemical inhibitor of ETAR signaling. Notably, the inhibition of ETAR signaling in vivo demolished the ability of IL27 to promote angiogenesis (Figure 4E, 4F).

### IL27 enhances papilloma formation in the skin and proliferation of mutated stem cells

**Figure 5:**
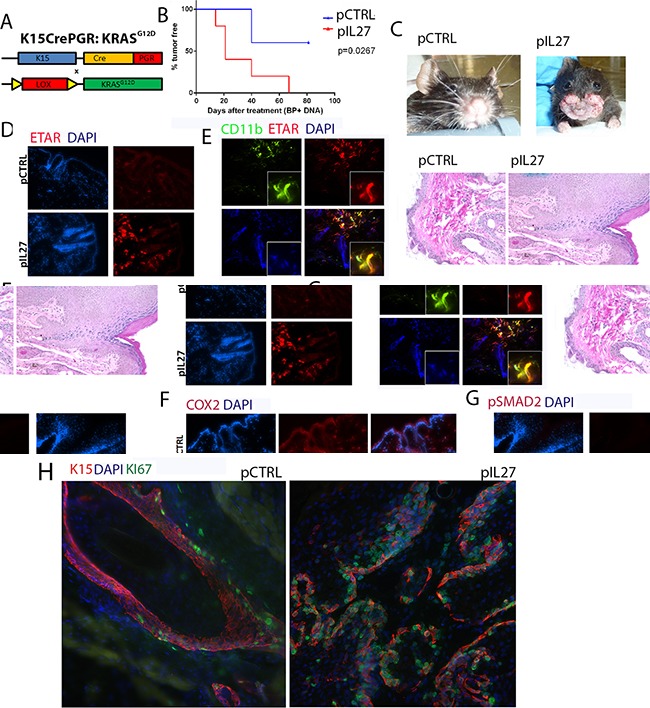
IL27 enhances papilloma formation in the skin and proliferation of mutated stem cells **A.** Schematic representation of the generation of the K15:KRAS^G12D^ mouse. **B.** Gene therapy administration of plasmid IL27 and topical administration of BP treatment enhances visible papilloma induction in K15:KRAS^G12D^ mice when compared to control plasmid (pCTRL) and BP. Induction of mutant KRAS in 6-8 week old K15-KRAS^G12D^. N=3-5 mice per group; experiment was repeated twice **C.** Macroscopic phenotype and HE of papilloma appearance in K15:KRAS^G12D^ mice. **D, E, F.** Immunofluorescence analysis showing increase in ETAR staining (D), ETAR-positive CD11b-positve cells (40X) (E) and COX2 levels (F), were increased in the skin of K15-KRAS^G12D^ 3 weeks post IL27 treatment via gene therapy when compared to control (200X). **G.** Immunofluorescence analysis showed pSMAD2 levels were increased in the papillomas from IL27- treated K15-KRAS^G12D^ when compared to control plasmid treated mice (Skin tissue was analyzed 10 weeks post KRAS induction; pictures were taken at 200X). **H.** Immunofluorescence analysis showed higher number of Ki67-positive K15 stem cells in the papillomas of mice treated with IL27 when compared to control plasmid (Skin tissue was analyzed 10 weeks post KRAS^D12D^ induction, 200X). Nuclear staining was visualized with DAPI.

The observation that IL27 promotes skin angiogenesis via ETAR provoked further investigation into the effect of the premalignant niche on tumor initiation and growth. One possibility is that IL27-trained pre-malignant niche may affects mutated skin stem cells as cancer stem cells have been detected and described in many carcinogenesis models, including the skin [20–22]. Additionally, others have found that angiogenic factors and the vascular niche can regulate proliferation of these stem cells and tumor development in the skin [9]. Recently, lineage tracing revealed that most often K15 positive cells give rise to most papillomas in the DMBA/TPA model [23]. These studies suggest that IL27 drives ETAR-positive CD11b-positive cells to accumulate in the premalignant skin and may promote the proliferation of these stem cells that harbor the mutation. To address this question, mice carrying mutated KRAS (KRAS^G12D^) in the follicular stem cells (K15:KRASG12D) were treated with control or IL27 DNA plasmid and monitored for tumor incidence and growth. Indeed, in transgenic mice harboring the mutated KRAS in the K15+ follicular stem cells, IL27 promoted faster visible papilloma incidence and induced higher ETAR expression in the CD11b-positve cells in the pre-malignant skin (Figure 5A–5E). Interestingly, although benzoyl peroxide was applied to the back of the skin, we saw visible papillomas in the oral area. Histological examination of the skin in the back of the mice mainly showed only epidermal hyperplasia at the time the mice were required to be euthanized due to papilloma load on the oral/facial area. One possible explanation for fast tumor development in the oral/facial area is that aside from being exposed to the benzoyl peroxide due to grooming, this area undergoes more often mechanical stress. Because the tumor development was visual, the authors used this anatomical site to delineate papilloma tumor development.

COX2, a factor downstream from ETAR which is also an important molecule in upregulating angiogenesis and increasing the levels of activated KRAS [24], was highly upregulated by IL27 in the premalignant skin (Figure 5F). TGFβ signaling, a marker of cells going through tissue regeneration [25], was increased in papillomas derived from IL27-treated mice, as determined by the phosphorylation of the effector protein pSMAD2 (Figure 5G). More importantly, the number of proliferating K15-positive stem cells was increased in papillomas derived from IL27-treated mice when compared to control-treated mice (Figure 5H). In summary, these data suggest that IL27 creates a pre-malignant niche that enhances the proliferation of mutated bulge stem cells.

### IL27 enhances papilloma formation in K15-KRAS^G12D^-driven mouse model via ETAR signaling

**Figure 6:**
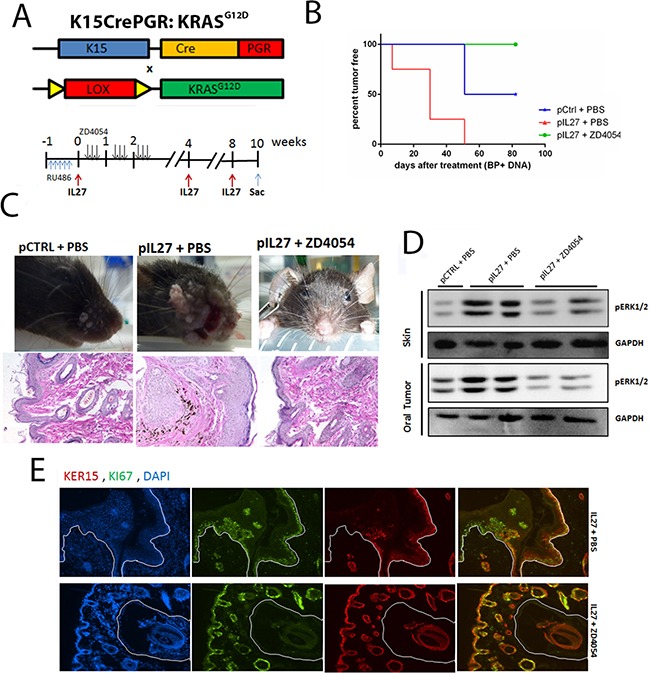
IL27 enhances papilloma formation and proliferation of mutated bulge stem cells via ETAR **A.** Model describing the experimental design of the ZD4054 administration in the K15:KRAS^G12D^ mouse. **B.** Gene therapy administration of plasmid IL27 and topical administration of BP treatment enhances visible papilloma induction in K15:KRAS^G12D^ mice when compared to control plasmid (pCTRL) and BP while inhibitions of ETAR via ZD4054 reverses IL27's ability to promote papillomas. Induction of mutant KRAS in 6-8 week old K15-KRAS^G12D^. N=3-4 mice per group; experiment repeated twice. **C.** Macroscopic phenotype and HE of papilloma appearance in K15:KRAS^G12D^ mice treated with control DNA, IL27 and vehicle control, or IL27 and ZD4054. **D.** Western blot showing that inhibition of ETAR signaling reverses IL27's ability to increase pERK1/2 levels in both pre-malignant skin and oral tumors (each lane is independent mice). **E.** Immunofluorescence analysis showing reduced number of Ki67-positive K15 stem cells in the papillomas of mice treated with IL27 and ZD4054 when compared to IL27 plasmid and vehicle control. (D, E) Skin tissue was analyzed 10 weeks post KRAS activation at 200X. Nuclear staining was visualized with DAPI.

The next step was to delineate whether the ET1/ETAR pathway is the missing link between the IL27-driven pre-malignant skin and KRAS activation in the K15-positive cells. To address this question, we used the chemical inhibitor ZD4054 which was previously shown to inhibit the ligand binding to ETAR [26]. As expected, mice treated with ZD4054 were mostly resistant to papilloma formation during the duration of the treatment (Figure 6A–6C).

The next issue necessary to understand is how the pre-malignant milieu created by IL27 and reversed by an ETAR inhibitor promotes the proliferation of the mutated follicular stem cells. Recent studies showed that the pro-inflammatory milieu can serve as an activator of KRAS by promoting higher levels of GTP-bound KRAS in the pancreas which leads to activation of mitogen-activated protein kinases, such as pERK [6, 24]. In fact, activation of pERK was higher in the premalignant skin of IL27-treated mice when compared to the control-plasmid treated mice, levels which were reversed in the presence of the ETAR inhibitor (Figure 6D). pERK was mainly up regulated in the stem cells of the skin (Supplementary Figure S8). Additionally, higher levels of active KRAS-GTP were found in the IL27-treated mice when compared to the control-treated mice, levels which were again reversed in the presence of ETAR inhibitor (Supplementary Figure S9). More importantly, the number of proliferating K15-positive stem cells in the skin was decreased in the presence of ETAR inhibitor when compared to IL27-treated mice (Figure 6E). In summary, IL27 can promote pre-malignant skin markers and papilloma formation via upregulation of ETAR-positive CD11b-cells in the skin.

## DISCUSSION

Inflammation can promote oncogene activation and subsequently lead to tumorigenesis. Here we establish a new concept in placing the inflammatory cues such as IL27 are at the forefront in regulating the establishment of pre-malignant niche, angiogenesis, oncogene activation, stem cell proliferation, and ultimately skin tumor formation, by stringing all these processes together via IL27- mediated induction of ETAR-positive CD11b cells.

### IL27 as a pro-tumor cytokine

A key finding of this study is the tumor promoter activity of IL27 in the skin, and this discovery is surprising given that previous studies have attributed to IL27 anti-tumor activities mainly via eliciting an anti-immune response [11] [27–33]. Furthermore, in vitro studies suggest that IL27 promotes accumulation of Th1 response in keratinocytes via secretion of CXCL10 [34]. On the other hand, endogenous IL27 has been related to features of tumor progression [35]. Other reports show an increase in IL27 in the sera of breast cancer patients, a high correlation between IL27 expression and the angiogenesis molecule VEGF, and a positive association with IL27 and clinical stage of breast cancer patients [35]. Furthermore, tumors transplanted in mice with a conditional knockout of IL27p28 in DC have reduced number of infiltrating T regulatory cells [36]. These functions are attributed to IL27 but not IL30, the p28 subunit of IL27 which can function independently of IL27, because reconstitution with IL27 but not IL30 promotes tumor growth in an IL27R-dependent matter [14, 36]. Meanwhile, in the study described here, it seems that IL27 promotes skin tumor initiation via propagating a pro-inflammatory microenvironment, which is consistent with another report demonstrating that IL27 would exacerbate skin inflammation in an experimental model of psoriasis-like skin inflammation induced by imiquimod [37]. Furthermore, the pro-inflammatory properties of IL27 in the skin sync well with its ability to exacerbate other inflammatory diseases such as colitis and arthritis [38, 39].

### IL27 promotes skin tumorigenesis via ETAR-positive CD11b cells

One key finding from this study is that IL27 promotes skin tumorigenesis via ETAR-positive CD11b cells. Two lines of evidence support this claim: first, IL27-treated CD11b^−/−^ mice are 4 times less likely to develop papillomas and promote angiogenesis in the pre-malignant skin when compared to control, and second, ETAR is mainly upregulated in CD11b-positive cells and ETAR depletion via a chemical inhibitor significantly delayed IL27-dependent papilloma development in the K15-driven oncogenic KRAS model. The argument that IL27-drived skin tumorigenesis is dependent on ETAR signaling other than CD11b cells is unlikely (for example vessel cells), as in absence of CD11b positive cells, IL27 cannot promote skin tumorigenesis although ETAR signaling or angiogenic signaling is not perturbed directly.

One of the main cell types involved in IL27's ability to promote skin tumorigenesis are CD11b cells. Such discovery of the connection between IL27 and CD11b-positive cells bodes well with recent studies showing that IL27 directly induces differentiation in hematopoietic stem cells [40]. More specifically, IL27 can promotes expansion of only Lineage^−^Sca1^+^cKit^+^ (LSK) cells, especially long-term repopulating HSC and myeloid-restricted progenitor cells during emergency myelopoiesis such as malaria infection [41].

One explanation for CD11b positive involvement in skin carcinogenesis is that CD11b^−/−^ mice have a reduced number of myeloid cells because of defects in attachment and trafficking of leukocytes to the sites of inflammation [19]. Alternatively, CD11b is part of the CR3 complex which binds complement component iC3b and many other ligands [42]. The CR3 complex can also bind many other molecules such as uPAR, e-selectin, complement factor H, etc, which might regulate ETAR [43, 44]. Additionally, decreasing myeloid mobilization may also decrease a major source of IL-27 in the skin microenvironment. However, this possibility may be unlikely because electroporation induces robust levels of IL27 in the serum in 400 pg/ml, and such levels may compensate loss of endogenous levels from myeloid cells. One final plausible explanation is that CD11b^−/−^ mice have a lower number of immunosuppressive myeloid-derived suppressor cells, a well-recognized pro-tumor cell population in many tumors including the skin [45].

Another interesting finding of this study is the involvement of ETAR in the establishment of the premalignant niche in cutaneous malignancies, and this IL27-induced ETAR upregulation in CD11b cells is an early event in this process. ETAR is induced as early as 72 hours, but more sustained levels are seen around 3 weeks post IL27 treatment. ETAR can sustain the pre-malignant niche by inducing angiogenesis in the pre-malignant skin. ETAR involvement in promoting inflammation is expected as previous reports show that the ET1/ETAR pathway can sustain the pre-metastatic niche in the lung via upregulating pro-inflammatory cytokines or chemokines such as IL6, CCL2, and COX2 [26, 46]. Additionally, concurrent with our results, others have also shown that inhibition of ETAR can decrease vessel density [26]. While no direct connection between IL27 and Endothelin pathway has been reported in the literature, we are the first to describe this relationship

ETAR is upregulated by IL27 mainly in CD11b-positive cells while its expression in epithelial cells in the pre-malignant niche is undetectable (Figure 5). Others have shown that this receptor can be expressed in SCC or in vessels [47]. However, administration of the chemical inhibitor was done at the same time of the induction of the KRAS, when ETAR is expressed exclusively in the CD11b positive cells but is undetectable in the epithelial cells. In the K15-KRAS^G12D^ model, zibotentan can reverse IL27-driven changes in the pre-malignant niche and initiation of papillomas (Figure 6), suggesting that the contribution of ETAR signaling in epithelial cells is unlikely to explain the ETAR-dependent pro-tumor effects of IL27.

### IL27's establishment of the pre-malignant niche and tumor formation

An important consequence associated with IL27 in the skin carcinogenesis is the increase in vessel density (Figure 2). In this skin, the vasculature niche in the skin plays a crucial role in initiation and growth of SCC and increasing cancer stem cell population in the tumors. More concretely, the deletion of VEGFR1 or VEGFα in epithelial cells delays papilloma formation [17, 48, 49]. Likewise transgenic overexpression of VEGF in the keratinocytes accelerates skin tumor development [49]. Aside from promoting tumor growth, the vasculature niche can also promote stemness of SCC tumors, as these CSC often reside in perivascular niches [9]. While the link between angiogenesis and the stem cell niche has been established, this report uncovers the upstream mechanism that governs the vascular niche and identifies the key role of IL27 in establishing the vasculature niche in the skin by upregulating the Endothelin A receptor (ETAR) CD11b cells. Indeed, inhibition of ETAR signaling in two different models, DMBA/BP and K15-KRAS mouse models, halts IL27-mediated upregulation of CD31-positive cells, which is consistent with other studies linking ETAR and angiogenesis [26]. Yet, if and how ETAR affects stem cell proliferation is not well-understood. At this point we speculate that ETAR has a dual role in promoting stem cell proliferation in the skin. The first role could be through promoting the vasculature niche via mechanisms described by Blanpain and colleagues [9]. The second role could be through inducing the pro-inflammatory pathways such as COX2 in the microenvironment, as COX2 amplifies K-RAS^G12D^ to pathological levels in the pancreas [24, 50, 51]. In the K15-KRAS^G12D^ model used, one mechanism that is associated with the increased proliferative abilities of these stem cells is the amount of activated GTP-bound KRAS in the skin (Supplementary Figure S9) and the activation of pERK1/2 in the follicle (Supplementary Figure S8). Inflammatory cues can indeed increase the amount of activated KRAS in the pancreas [24, 50, 51]; however, this report is the first to detail the pre-malignant inflammatory cues (IL27/BP) in the skin which increase activated KRAS and proliferation of K15-positive stem cells.

## MATERIALS AND METHODS

### Transgenic mice

To generate K15:KRASG12D, B6;SJL-Tg(Krt1-15-cre/PGR)22Cot/J were bred to B6.129S4-Krastm4tyj/J. Mice positive for both KRAS and Cre were used for these studies. To activate KRAS, strictly only 6-8 week old mice were treated with 2 mg/day of RU486 diluted in acetone (Cayman) for 5 consecutive days. Zibotentan (ZD4054, Selleck Biologicals), 5mg/kg/mouse or vehicle control was given IP to mice three times a week, diluted at 100 ul of saline per mouse for the duration of the study. The first dose of ZD4054 was given 24 hours prior to the IL27 treatment. Mice were treated once a month with 6ug of DNA injected in the rear tibialis muscle followed by electroporation as previously described (23). CD11b^−/−^ (B6.129S4-Itgamtm1Myd/J), JH^−/−^ (B6.129P2-Igh-Jtm1cgn/J^−/−^, and wildtype C57Bl/6 were purchased from the Jackson laboratories and NCI respectively. IL27RA^−/−^ mice in a C57Bl/6 background were previously donated by Dr. Frederic de Sauvage (Genentech). All mouse experiments were approved by MD Anderson Cancer Center Institutional Animal Care and Use Committee.

### DMBA and Benzoyl Peroxide (BP) mouse treatment

Once mice reached 7-8 weeks old, mice were shaved with an electrical clipper 3-4 days prior to the application of 100 ul of 0.1% w/v of DMBA dissolved in acetone applied topically [52]. One week post DMBA application, mice were treated with the promoter benzoyl peroxide topically (20mg/mouse dissolved in 200 ul of acetone) twice a week for the duration of the study. Papilloma incidence was monitored weekly. Mice were treated once a month with 6ug of DNA injected in the rear tibialis muscle followed by electroporation. DNA was injected at the same time as the benzoyl peroxide treatment (1 week post DMBA). The gene clones used in this study include pIL27, and pCTRL. IL27 is a generous gift from Dr. Masatoshi Tagawa (Chiba Cancer Center Research Institute, Japan). From this plasmid, both subunits of IL27 were subcloned to our control vector pVC1157 (pCTRL) to yield the pIL27 encoding gene construct.

### Immunohistochemistry and immunofluorescence detection

Data shown are representative of at least three different mice per group. Briefly, frozen sections were fixed in acetone for 10 minutes in -20C, blocked in 3% fish gel for 30 mins, and stained with primary antibodies diluted in 3-5% fish gel overnight at 4C. Next, sections were washed three times in PBS, blocked in 3-5% fish gel solution at room temperature for 15-20 minutes, and incubated for 1 hour at room temperature in secondary antibody diluted in 4.5% fish gel. Briefly, these antibodies were used: ETAR (1:800, Fisher), ETBR (1:800, Fisher), F4/80 (1:100, AbD Serotec), CD31 (1:400,BD Pharmingen), Keratin 15 (1:400, GeneTex), pSMAD2 (1:50, Cell Signaling), CD34 (1:800, BD Pharmingen), CD4 (1:400, AbD Serotec), CD8 (1: 200, AbD Serotec), COX2 (1:200, Oxford Biomedical), pERK1/2 (1:50, Cell Signaling), SMA (). PDGFA (1: 100; Santa Cruz), PDGFB (1: 100; Santa Cruz), PDGFR-α-PY720 (1: 100; Santa Cruz), PDGFR-β-PY751 (1:100, Cell Signaling), Ki67 (1:800, eBioscience), ET1/2/3 (1: 100, Santa Cruz) anti-mouse IgG- Alexaflour 488 (1:1600, Life Technologies), anti-mouse IgM–Alexaflour 488 (1:1600, Life Technologies), anti-rat IgG-Alexaflour 488 (1:800, Life Technologies), anti-rabbit IgG- Alexaflour 555 (1:800, Life Technologies). Pictures were acquired using Nikon Eclipse Ti microscope. To detect the human IL27RA in the tissue array via immunohistochemistry, we used SK802a skin tissue array purchased from US Biomax Inc. Briefly, paraffin-embedded unstained slides from patients were deparaffinized, heat-induced antigen retrieval in pH 6.0 citrate buffer for 40 min in the steamer. Monoclonal IL27RA antibody at 0.7 ug/ul was diluted 1:100 in goat/fish gel/serum blocking buffer and incubated overnight at 4C. The next day, tissue was washed, blocked for 15 min in blocking buffer, and incubated with anti-mouse IgG-Biotin (1:400 diluted in blocking buffer) for 1 hour at room temperature, followed by 30 min via ABC enhanced system (Vectastain Kit).

### Western blot

Briefly, tissue from the treated skin of mice or tumor was snap frozen in liquid nitrogen. Tissue was homogenized in lysis buffer, and supernatants were quantified for protein amount. 160 ug of cell lysate was loaded in 10% SDS-PAGE, transferred to a nitrocellulose membrane and incubated with primary antibody overnight at 4°C (1000x dilution for anti-pERK1/2 (Santa Cruz), 2500x anti-GAPDH, 5000x anti-mouse IgG-HRP (Santa Cruz). To detect 1000x anti-RAS (Millipore), lysates were loaded in a 15% gel.

### RAS activity assay

To determine the levels of GTP-bound RAS that is able to bind Raf, we used a Raf-pulldown assay kit as previously described in detail by Huang et al [50]. Briefly skin sample were homogenized is ice using a lysis buffer containing 25 mm 4-(2–hydroxyethyl)-1-piperazineethanesulfonic acid (pH 7.5), 1% Igepal CA-630, 150 mm NaCl, 0.25%sodium deoxycholate, 10% glycerol, 25 mm NaF, 10 mm MgCl2, 1 mmethylenediaminetetraacetic acid, 10 μ g/ml aprotinin, 10 μ g/ml leupeptin and 1 mM sodium orthovanadate. Lysates were spun down at maximum speed for 10 minutes at 4C. Supernatants were quantified and diluted at 1mg/ml in lysis buffer. 1 ml of lysate was incubated for 30 min at 4C with 10 μL of Raf-coated agarose beads. The beads were washed with lysis buffer 4 times. During each wash, the beads were spun down for 4 seconds and the supernatant was discarded. The beads were boiled and total levelsof RAS were visualized by western-blot analysis.

### ImageJ quantification via color deconvolution

For the quantification of IL27RA receptor in human patient samples, we used ImageJ program developed by NIH with Color Deconvolution plugin. Briefly, color deconvolution allows for the isolation of the DAB stain (which represents IL27RA positive cells) from hematoxylin staining, which represents the nuclei. Next, the brown image was converted into binary image (black and white, where black represents DAB stain and white represents non-DAB stain). Values are calculated as mean of black and white values for each area; higher mean value means higher number of black values, and therefore, higher % of DAB-positive area. Next, we took at least 3-6 independent areas from each patient sample from non-epithelial area, and averaged these values to calculate a representative value for that sample.

To calculate corrected total cell fluorescence (CTCF), we used the method described by Burgess *et. al*. [53]. Briefly, the following formula was used: CTCF= Integrated Density – (Area of Selected cell X Mean Fluorescence of background reading). To calculate CD31 positive area, fluorescence signal was converted to binary image. % area was calculated by dividing the CD31 positive stained area to total skin area.

### Bone marrow chimeras

8 weeks old IL27RA or WT mice were lethally irradiated with 900Gy. Within the same day, these mice were divided in groups and were reconstituted with 1*10^7^ bone marrow cells obtained from C57Bl/6 or IL27RA^−/−^. These bone marrow cells were suspended in 100 ul of PBS and were injected via IV into mice. Irradiated mice were kept for 2 weeks in water containing antibiotics. 8 weeks post-irradiation, these mice were confirmed by FACS analysis for different immune cell population for successful reconstitution. DMBA/BP treatment was initiated at 6 weeks post-irradiation.

### Genotyping

DNA was extracted from ear tissue, and PCR was performed using the primer sequence described below. For K15, the presence of Cre gene was confirmed via using forward sequence CCATCTGCCACCAGCCAG and reverse sequence TCGCCATCTTCCAGCAGG. To determine the presence of KRAS^G12D^ mutant, JAX suggested primers sequence were used.

### IL27RA monoclonal antibody production

Recombinant human exogenous IL27RA-Fc (rhIL27RA) (R&D Systems) was used as an antigen for monoclonal antibody production by the Monoclonal Antibodies Facility Core at MD Anderson Cancer Center. Anti-IL27RA titer was determined using ELISA assay. Briefly, rhIL27RA was used as solid antigen in ELISA screening. Hybridomas yielding antibody clones that showed higher optical density at lower dilutions were selected for further validation, which includes flow cytometry, western blot analysis, and immunohistochemistry. This series of screening led to the selection of clone 237 for this study.

### Flow cytometry analysis

Isolated bone marrow cells from 3 month old mice and were blocked for non-specific staining in 2% BSA and with anti-CD16 (5 μ g/mL) for 20′ at 4°C. Afterwards, cells were stained in 2% BSA with anti-CD4-Violet421 (1:100, Pharminogen), anti-CD8-PE-Cy7 (1:100, Biolegend), anti-F4/80- Fitc (1:100, eBiosciences), anti-CD3-e450 (eBiosciences), anti-NK1.1- PE-Cy7 (eBiosciences), anti-CD19-Violet421 (1:100, Biolegend) and anti-B220-PerCP (1:100, Biolegend), anti-CD11c-Violet 421 (1:100, Biolegend), anti-MHCII-PerCP (1:100, Biolegend). At last, the cells were washed twice in 2% BSA and analyzed by flow cytometry using Attune (Invitrogen), and FlowJo.

### Statistical analysis

Kaplan-Meier survival with Mantel-Cox test was used to analyze incidence of papilloma in Figure 1B-D. Student's T test was used to analyze the differences in Figure 1E, 2 and 3. GraphPad Prism for Windows was used to prepare and analyze the graphs (GraphPad Software).

### Study approval

This study was approved by the institutional review board at MD Anderson Cancer Center.

## SUPPLEMENTARY MATERIALS FIGURES


