# Supplementary material for: IL27 controls skin tumorigenesis via accumulation of ETAR-positive CD11b cells in the pre-malignant skin
Source: Oncotarget. 2016 Oct 12;7(47):77138–51. doi: 10.18632/oncotarget.12581 (PMC5363575; doi:10.18632/oncotarget.12581)
Supplement: Supplementary file 1 [file oncotarget-07-77138-s001.pdf]

IL27 controls skin tumorigenesis via accumulation of ETAR-positive CD11b cells in the pre-malignant skin

SUPPLEMENTARY FIGURES

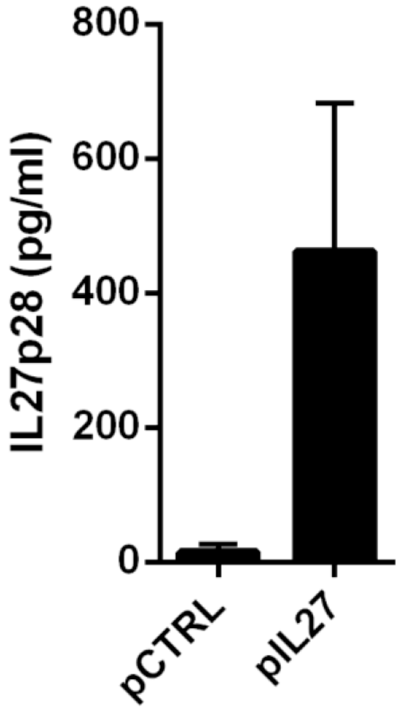

Supplementary Figure S1: Electroporation gene delivery is an efficient method to deliver IL27 systemically. Plasmid expressing both subunits of IL27 or empty vector were injected in the rear tibialis muscle and electroporation was applied to the rear tibialis muscle. 48 hours post electroporation gene therapy, the concentration of IL27 protein in the serum was measured via ELISA. N=4.

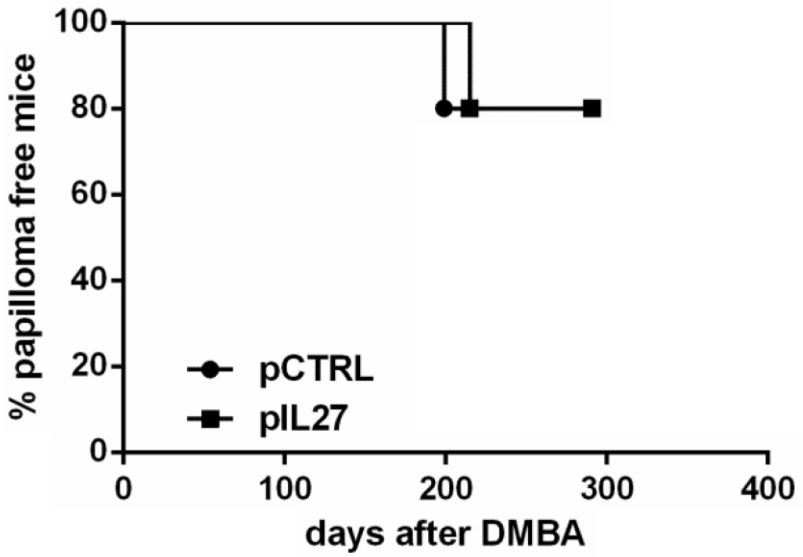

Supplementary Figure S2: IL27 signaling through IL27RA receptor is needed to promote papilloma initiation. IL27RA$^{-/-}$ mice are resistant to papilloma formation and have similar papilloma incidence by administration of either IL27 or control plasmid DNA via gene therapy. N=5.

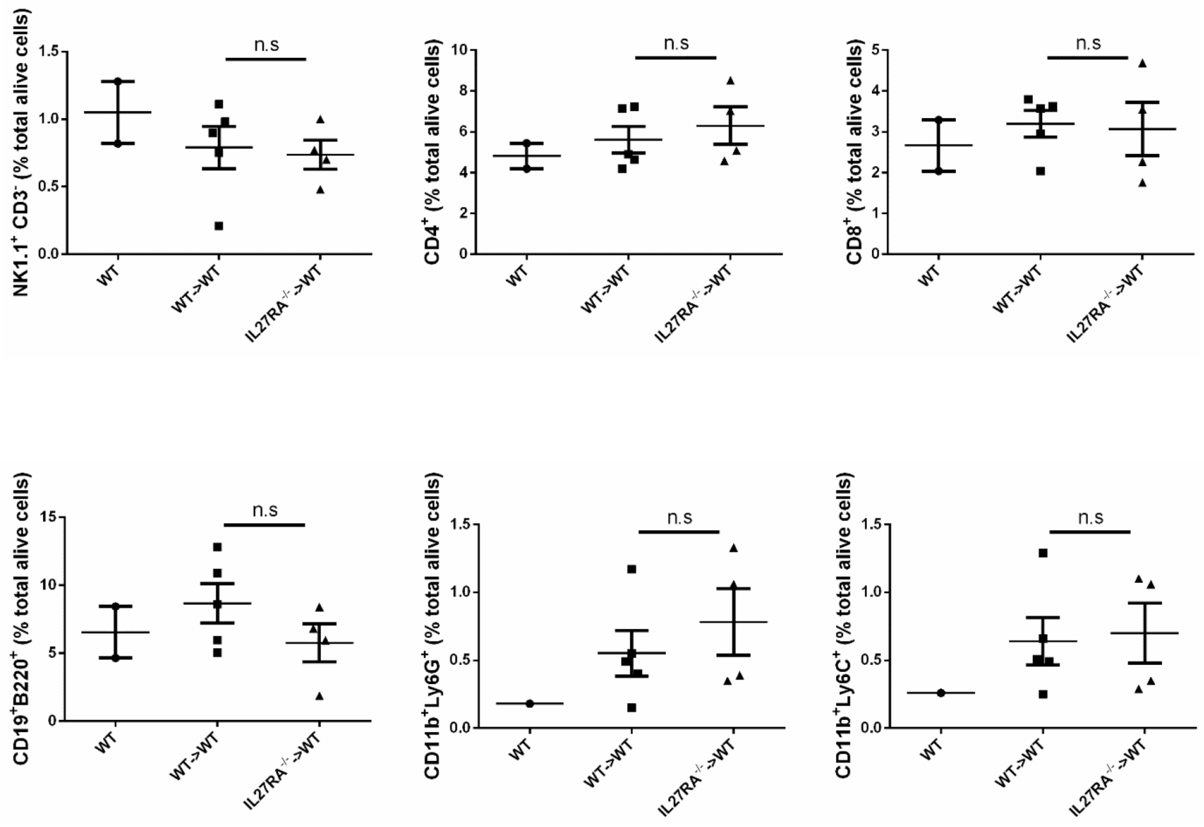

Supplementary Figure S3: Reconstitution efficacy in the peripheral blood 8 weeks post bone marrow transfer. Lethally irradiated wildtype mice were reconstituted with bone marrow derived from either wildtype (WT->WT) or IL27RA$^{-/-}$ (IL27RA$^{-/-}$->WT) mice. 8 weeks post-irradiation, the percentage of different immune cells in the peripheral blood were enumerated via flow cytometry. N=5/group.

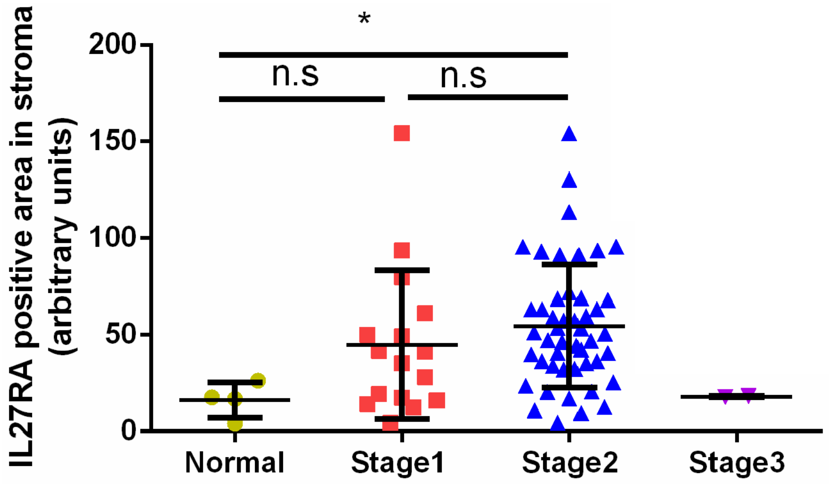

Supplementary Figure S4: IL27RA positive cells had no correlation to disease stage. Quantification and representative photomicrographs of IL27RA positive cells in non-epithelial (stroma) compartment of skin derived from normal patients, or patients with SCC at different stages. Pathology report provided by US Biomax.

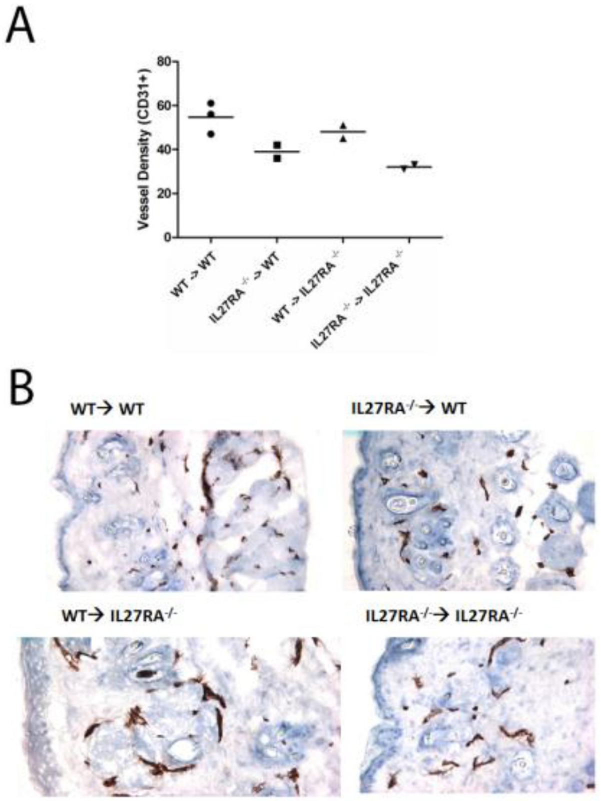

Supplementary Figure S5: IL27 signaling through IL27RA receptor in bone marrow cells is needed to promote angiogenesis. A. Enumeration of vessel density and representative photomicrographs **B.** of skin tissue after bone marrow transfer as depicted in the Figure 1.

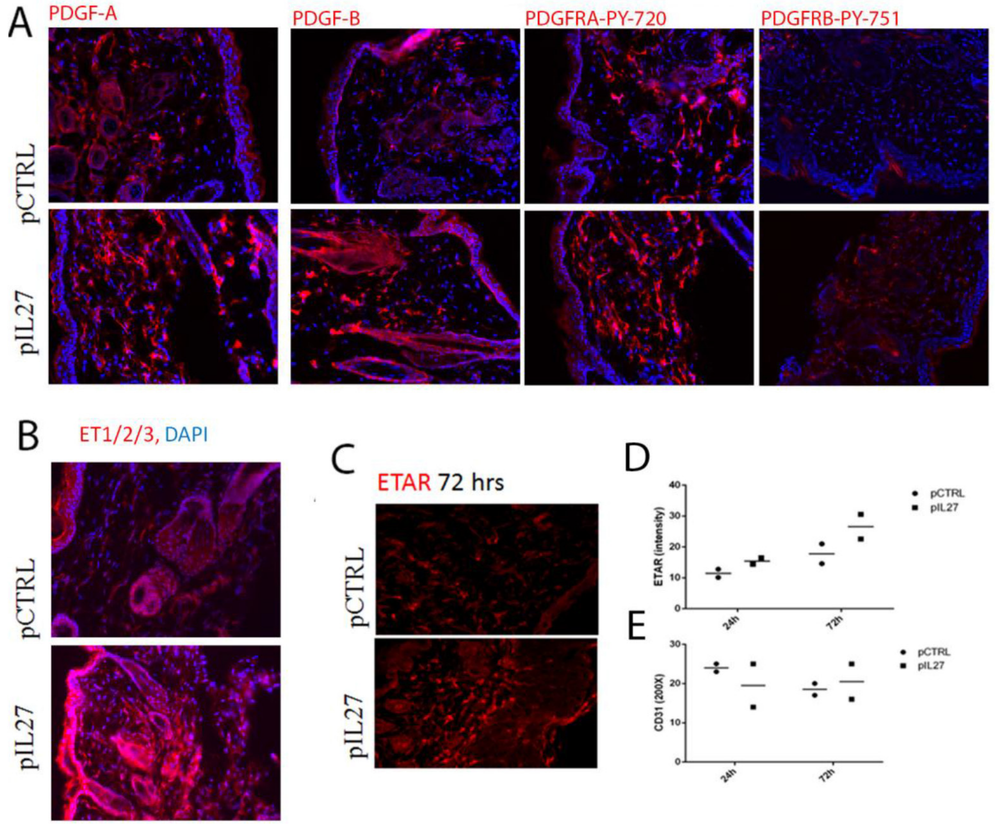

Supplementary Figure S6: IL27 activates pro-angiogenic molecules in the premalignant skin microenvironment. **A.** Representative pictures of frozen pre-malignant skin sections harvested 8 months post DMBA treatment from wildtype mice treated with either control plasmid-encoding DNA or IL27 that were analyzed by immunofluorescence staining of Endothelin Receptor A, PDGFA, PDGFB, PDGFRA-PY720, and PDGFRB-PY-751 (red signal). Nuclear staining was done with H33342 (blue signal). Pictures were taken at 200X. **B.** Immunofluorescence analysis showed that IL27 increases Endothelin levels in the skin. Representative picture of frozen skin tissue was analyzed 8 months post treatment with DMBA/BP for Endothelin 1/2/3. **C, D, E.** Immunofluorescence analysis showed that IL27 increases Endothelin A receptor levels were raised in the skin as early as 72 hours. Representative pictures of frozen pre-malignant skin sections harvested 24 or 72 hours post treatment with either control plasmid-encoding DNA or IL27 that were analyzed by immunofluorescence staining of Endothelin Receptor A (C, D) and vessel density (E).

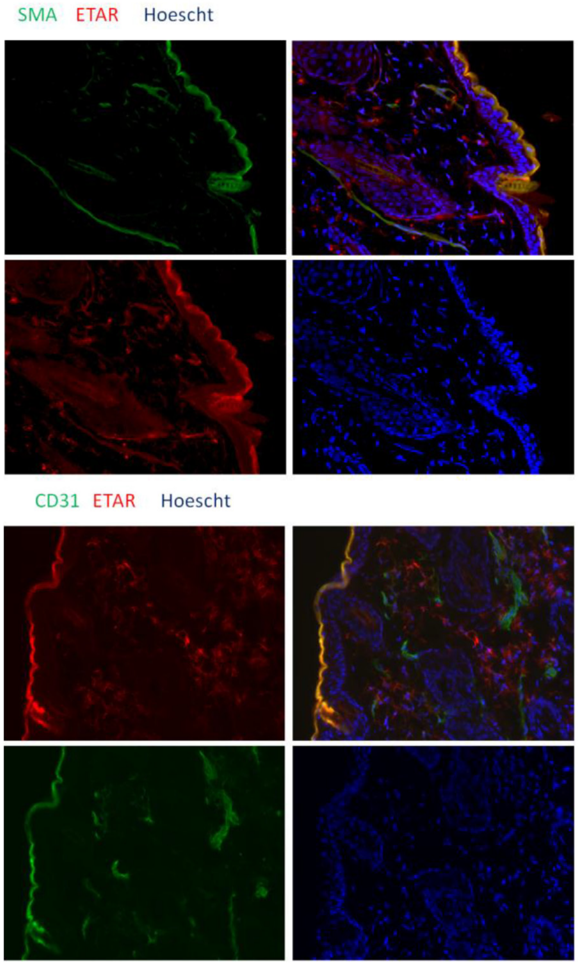

Supplementary Figure S7: ETAR is not upregulated in endothelial cells. A. SMA and ETAR or CD31 and ETAR **B.** immunolocalization in the skin tissue of mice treated with either IL27 or control plasmid via gene therapy.

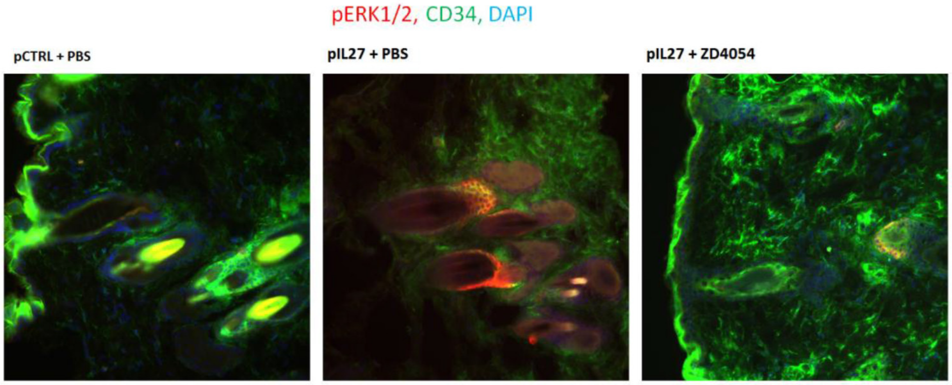

Supplementary Figure S8: pERK1/2 and CD34 immunolocalization in the hair follicle of mice treated with either IL27 or control plasmid via gene therapy in K15-KRAS^{G12D} mice. Nuclear staining was visualized with DAPI. Skin tissue was analyzed 10 weeks post KRAS induction, 200X.

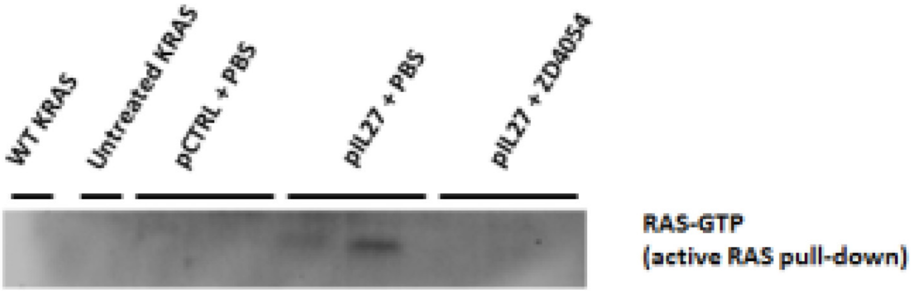

Supplementary Figure S9: IL27-induced active KRAS is reversed in absence of ETAR signaling. Active RAS levels was measured by a Raf pull-down assay from the skin lysates of wildtype, or K15-KRAS mice left untreated, or treated as indicated in the Figure. Each lane represents an independent mouse.
